# Technology as the key to women’s empowerment: a scoping review

**DOI:** 10.1186/s12905-021-01225-4

**Published:** 2021-02-23

**Authors:** April Mackey, Pammla Petrucka

**Affiliations:** University of Saskatchewan, 100-4400 4th Ave, Regina, SK S4T 0H8 Canada

**Keywords:** Information and communications technology, Impact, Women empowerment, Scoping review

## Abstract

**Background:**

Information and communications technologies (ICTs) have empowered people to communicate and network at a global scale. However, there is lack of in-depth understanding of the use of ICTs for women's empowerment. This study examines how the concept empowerment is defined, utilized and measured in research studies, the existing evidence on the use of ICTs for women’s empowerment and the gaps in knowledge at the global level.

**Methods:**

The authors’ conducted a scoping review using the Arksey and O’Malley methodology. The search identified papers from ten databases, including Scopus, Embase, ABI Inform, Soc Index, Sociological Abstracts, Gender Studies, Springer Link, PsychInfo, Science Direct, and Academic Search Complete over the period of 2012–2018. Search criteria included articles that focused on women’s empowerment and utilized technologies as interventions. Out of a total of 4481 articles that were initially identified, 51 were included.

**Results:**

Technology played a variety of roles in supporting the development of women’s capacities and resources. Results revealed the use of ICT interventions in the overarching areas of outreach (e.g., health promotion), education (e.g., health literacy opportunities), lifestyle (e.g., peer coaching and planning), prevention (e.g., screening opportunities), health challenges (e.g., intimate partner violence apps), and perceptions of barriers (i.e., uptake, utilization and ubiquity to ICTs for women). Despite the positive use of technology to support women in their daily lives, there was a lack of consensus regarding the definition and use of the term empowerment. The concept of empowerment was also inconsistently and poorly measured in individual studies making it difficult to determine if it was achieved.

**Conclusion:**

This scoping review provides a comprehensive review of current and emerging efforts to use ICTs to empower women. The findings suggest a need for collaborative efforts between researchers, program implementers and policy makers as well as the various communities of women to address the persistent gender disparities with respect to ICTs.

## Background

The term women’s empowerment emerged in the 1970s in response to the need for social justice and gender equality [1, 2]. As the term evolved in the 1990s, it was increasingly applied to women who were oppressed and lacking the freedom of choice and action to shape their lives, as well as to discuss women’s participation across multiple sectors in society. More recently it has been used as an outcome and a goal to be achieved is to balance the scales of gender equality and equity. For this research, the definition used regarding women’s empowerment is a process by which women who have experienced oppression acquire the ability to make autonomous and strategic life choices based on their personal priorities. Empowerment is achieved when a woman has the resources, agency, and capabilities to execute decisions on matters of importance [3, 4].

Globally women are more likely to experience less favourable social determinants of health (such as over-representation of women in low-paying, insecure employment; lower education and literacy levels amongst rural and immigrant women) than their male counterparts. Women carry the bulk of responsibility for raising children and meeting household obligations, which, globally, contribute to this continued disadvantage [5–7]. Due to a lack of affordable and quality daycare, women are over-represented in part-time work force, and often remain within low-income bracket [5–8]. Gender, as a social determinant of health, is influenced by the “gendered” norms of the roles, personality traits, attitudes, relative power, and influence that society ascribes to it [9, 10]. The transition from the Millennium Development Goals to the Sustainable Development Goals (SDGs) in 2015 saw the emergence of Target 5 which aims to “Achieve gender equality and empower all women and girls” (p. 20) [11]. A major SDG indicator supporting attainment of women’s empowerment is “enhancing the use of enabling technology by increasing the proportion of women and girls who have access” (p. 20) [11].

Information and communication technologies (ICTs) have catalyzed communication and networking between and among people on a global scale. However, as ICTs have become ubiquitous and grown in both type and access, a digital divide has emerged. This divide parallels gaps in social contexts, such as income and education, as those who use and benefit from access to technologies often have other resources more readily available [12]. This divide widens the inequity and inequality gaps based on gender, age, disability, or socioeconomic status [13, 14].

Women’s empowerment and ICTs have been the subject of global goals, discussions, and debates for many decades [15, 16]. Global discussions, such as the 1995 World Conference on Women: Beijing Declaration and Platform for Action, deliberated and advocated for the inclusion of women in the information society in order to fully achieve women’s empowerment in connection with ICT. In 2013, 200 million more men had access to the internet than women [17]. Women use ICTs much less frequently and intensely than men [18–21] In 2016, the International Telecommunications Union (ITU) stated that the percentage of women gaining access to ICT is actually decreasing—with women utilizing ICTs 11% less than men in 2013 and 12% less than men in 2016 [19]. The most recent 2018 report indicated that the overall proportion of internet usage for women was 12% lower than men [19].

The extant evidence lacks sufficient depth and detail as to exactly how ICTs are being used by women and why they use it less frequently. An important aspect of empowerment in the context of ICTs is gaining a clearer picture as to the type of technologies and technological interventions being used by women. Many authors agree that improved access to ICTs can assist in providing women with employment resources and opportunities that could narrow the gender wage gap, assist in making education and health information more accessible, contribute to the end of violence against women, and lead to women’s empowerment and leadership [15, 22–24].

The objectives of this research were to: determine how the concept of empowerment is defined, utilized, and measured in research studies; explore existing evidence regarding the use of ICTs as interventions towards achieving women’s empowerment; and explore the gaps in knowledge and research on this topic from an individual, community, and global perspective.

## Methods

This research involved a scoping review, which is methodologically similar to a systematic review, to provide a rigourous synthesis of existing evidence [25, 26] For the purpose of this study, the scoping review framework used was described by Arksey and O’Malley [27] as a five-step process with an optional sixth step. These steps include: (1) identifying the research question, as the starting point to guide the search strategy; (2) identifying relevant studies, which involved the development of a comprehensive search strategy to ensure accurate and complete results; (3) selecting studies, which involved developing a-priori inclusion and exclusion criteria that were revised throughout the review process, as familiarity with the evidence increased; (4) charting the data, which involved charting and sorting key material from the results into themes and trends; (5) collating, summarizing, and reporting the results, which involved presenting the results as a narrative; and (6) consulting with relevant stakeholders, which is contingent upon time and resource considerations. For the purposes of this research, the sixth step was not performed.

### Review protocol, team, and management

To ensure transparency, rigour, reproducibility, and consistency, protocols were developed prior to the start of the research, for the inclusion criteria, search strategy, and data characterization. This helped to ensure an unbiased approach to the search protocol and to enhance rigour [27]. These are available upon request. The scoping review was conducted by a team of individuals with multi-disciplinary capabilities in nursing, knowledge synthesis methodologies, and ICTs. The primary reviewers included the lead and co-authors, as well as one research assistant. In addition, a University librarian was consulted throughout the search term selection process to ensure completeness and accuracy of search terms as well as a comprehensive and complete search strategy.

Any and all potentially relevant citations identified throughout all stages were imported into EndNote™, a reference management software, where duplicates were removed by the program and then double checked, and manually removed by the lead author; the list of citations was then imported into a web-based electronic systematic review management platform, DistillerSR™. The screening for article relevance, up to the data extraction stages, were conducted using this software. Two reviewers (i.e., lead author and research assistant) were involved throughout the selection and analysis process to ensure consistency, adherence to the inclusion/exclusion criteria, relevance to the research question, as well as the categorization of data into themes and patterns. As part of this process, all articles were screened by the lead author and research assistant. Any discrepancies were brought forward to the co-author who made an independent decision whether to include or exclude the article.

### Review intent and scope

This was part of a broader study aimed at addressing the following question: What is the global impact of ICTs on women’s empowerment? The current review aimed to examine the concept of empowerment, while exploring the evidence on ICTs as interventions for achieving women’s empowerment at the individual, community, and global levels.

### Search strategy

The authors ensured identification of relevant and suitable publications by creating a search strategy protocol prior to retrieving evidence from a variety of sources. As per Arksey and O'Malley [27], the following avenues were reviewed as part of the search strategy: searching relevant electronic databases, reviewing reference lists of pertinent articles to identify additional sources, and manually searching key journals.

To ensure the search was comprehensive, the following databases, available through the University of Saskatchewan library, were searched on November 30, 2016 and updated on January 1, 2018: Scopus, Embase, ABI Inform, Soc Index, Sociological Abstracts, Gender Studies, Springer Link, PsychInfo, Science Direct, and Academic Search Complete. The COCHRANE Library was also searched for any relevant trials in the trial registry. Limits placed on the search included: English only, no book reviews, publications dated 2012–2017, and the protocol was pretested in Scopus and Soc Index using select key words including “women” and “empowerment” and “technology.” An illustration of the search term strategy is presented in Table 1.

**Table 1:** Search Term Strategy

Women Search Terms		ICT Search Terms		Empowerment Search Terms
*searched with OR	“AND”	*searched with OR	“ AND”	*searched with OR
Wom?n		Technolog*		Empower*
Female*		Information technolog*		Disempower*
Girl*		"information communications technolog*"		Barrier*
Maternal		"ICTs"		Enable*
		"social media"		Self concept
		mobile		Self efficacy
		handheld		Capacit*
		telehealth		Emancipat*
		computer		
		Smartphone		
		Digital		
		Internet		
		Telecommunication*		
		"world wide web"		
		Laptop		
		ICT4D		
		“web-based”		
		Iphone		
		Ipad		

*At end of word = truncation, any number of letters (e.g. capacit* will find capacity or capacities); ? at end of beginning of word is used to represent one or more other characters in a search term (e.g. wom?n will find women or woman)

Limits included: 2012–2017, English language, no books/book reviews

Search terms were drawn from the research question, as well as from lengthy discussions with the university librarian and expanded upon based on a cursory search of two databases. To determine the range and breadth of key terms, an initial limited search of two databases was conducted yielding several papers. These databases were determined in consultation with the university librarian and included Scopus and Gender Studies. These papers were then analyzed for similar keywords, definitions, analogies, and index terms that were relevant synonyms to the initial search words [28, 29]. These additional terms were added to a master list that informed the final search strategy. Specifically, for the term empowerment, keywords were chosen that could provide results that included a lack of empowerment as well, thus the inclusion of “barrier” and “disempower”. The other search terms came directly from key articles and databases and were demonstrated to be the most common variations on the term “empower”. An additional term that was used interchangeably with “empower” was “agency”, however, as this term is used more frequently in conjunction with organizations and not empowerment, it was removed from the search term list.

The ability of the electronic database search to identify all relevant primary research was verified by hand searching the reference lists of eight key peer reviewed articles and nine key electronic journals that were flagged through the initial test search as well as the main search. The journals were chosen based on their relevance to the research question as well as their scholarly nature. The initial three identified journals were: *Community Informatics*, *Gender and Development,* and *Journal of Women in Culture and Society.* Subsequent journals were identified and selected for a hand-search once the initial search was completed. These were*: Gender, Technology & Development*, *Computers in Human Behaviour*, *American Journal of Health Behaviour*, *American Journal of Public Health*, and *Women’s Health Issues*. These journals were then reviewed for additional articles potentially not identified through the database search; this included entering the general search into journal databases.

Additional grey literature was identified by hand-searching the websites of the Association for Computing Machinery Digital Library Journals and Conference Proceedings, the UN Women, Status of Women Canada, the United Nations Development Program, the International Center for the Research of Women, the Girls Action Foundation, the Information and Communications Technology Council, the ITU, and the International Development Research Center for primary research reports, guidelines, situation reports, and referenced publications that were not already included.

### Study selection: relevance screening and inclusion criteria

The focus of the study selection was locating published and unpublished academic articles, which may have any type of study design, including qualitative, quantitative, or mixed methods. The initial pool of results included a total of 4481 citations. An initial set of inclusion and exclusion criteria were developed a-priori to screen abstracts and titles of citations which were refined during each review of the pool of articles. Research articles were initially considered relevant if they included women’s empowerment and/or information and communication technology concepts in the title or abstract of the publication. Synonyms for these concepts were created in consultation with the librarian to ensure a robust search strategy for maximum location and inclusion of studies. Given the evolving nature of ICTs and their role in interventions, the authors wanted the articles to reflect a recent knowledge base, therefore the timeframe of 2012–2016 was chosen, which was later expanded to December 31, 2017 as the review progressed. The results were also filtered to include English only content.

### First screen: inclusion criteria

The inclusion criteria created for the first level of study selection were driven by the review topics, specifically, women, empowerment, and ICTs. According to the Joanna Briggs Institute (JBI) (2015), the inclusion criteria should be based on three themes, also known by the acronym of PCC: (a) participant description, (b) concept, which is likened to the phenomena of interest, and (c) context. The inclusion criteria used in the first level of selection were country of publication, date of publication (2012–2017), and the use of both of the following concepts in the title or abstract of the publication: women’s empowerment and/or information and communication technology. At this stage, the lead author looked for the presence of the key words in the title and/or abstract. The use of these keywords as inclusion criteria was designed to be intentionally broad to provide a sense of what publications linked the two concepts (i.e., women's empowerment and ICTs).

### First screen: study selection

On first review, the initial pool of articles was subjected to a staged process to ensure studies were selected that were relevant to the research question and met the inclusion criteria. Articles were first excluded based on duplication within the initial search results. This exclusion was conducted using the search tools feature within the electronic database, but also within the reference management program Endnote™ and then manually by the lead author. The inclusion criteria were applied to the title and abstract of the publication. Any title or abstract that did not meet the inclusion criteria was removed from further review and consideration. All articles excluded by the criteria were sent to the research assistant who confirmed the exclusion. Any disagreements or contradictions between the primary author and the research assistant were thoroughly discussed, with both parties having to agree to the inclusion before the publication could be added back into the pool of articles to move on to the next stage. Additionally, if an article could not be excluded based solely on the title or the abstract, the full article was reviewed for relevance to the research question and inclusion criteria. These latter two points did not prove to be an issue as there were no disagreements.

### Second screen

The remaining pool of articles was then reviewed a second time by applying a second level of inclusion criteria to the title as well as the abstract. It is common and encouraged as part of the scoping review process to generate increased cumulative familiarity with how concepts are presented within the evidence. This, in turn, informed the decisions that were made regarding the inclusion or exclusion criteria in the subsequent stage. Much of the articles after the first level of elimination included technology as a passive aspect of the study and not one that women actively participated in. It was important for the authors that the technological aspect of each study be an intervention that women could engage in towards building self-efficacy and capacity. This informs current gaps within the evidence that speak to how women are using technologies to support their empowerment. As such, this set of inclusion criteria focused on technology as an intervention and women as active participants in the study instead of just the word “women” found throughout the first set of criteria.

### Final screen

**Fig. 1:**
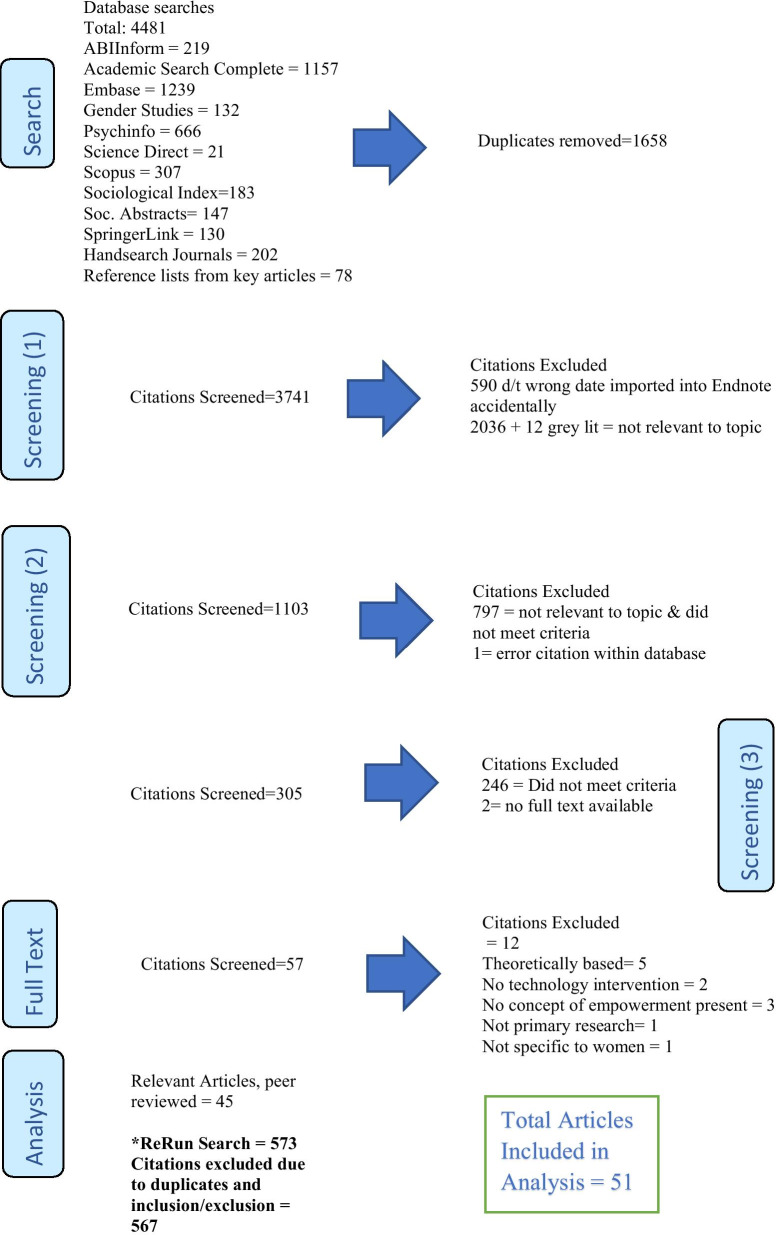
PRISMA Flow for Screening Process

For the final review of the full text articles, based on the content and findings in the scoping review process, an additional criterion was included. The authors wanted to explore how the social determinants of health informed and supported the concepts of women, empowerment, and ICTs. At this stage, it was noted which social determinants of health, if any, were present in each article. The list of social determinants based on the Government of Canada (2019) criterion was utilized as a reference for this portion of the process, such as employment and working conditions; income and social status; social supports and coping skills. The remaining 59 articles all had social determinants of health. A subsequent review resulted in 14 of the 59 articles being eliminated from consideration as they did not meet the inclusion/exclusion criteria. Rather than focus on a range of these determinants, the authors decided to include all 45 articles and to then review the implications of this finding in the analysis (Fig. 1).

### Re-run searches

Due to the extended time to conduct the review, the authors included re-run searches for each database up to January 1, 2018. A total of 573 articles were found in all 10 of the main electronic databases. Using the inclusion and exclusion criteria previously described all but six articles were eliminated through the first and second stages in the review process. The final total number of articles included in the analysis was 51.

### Study characteristics, extraction, and charting

The final step in the Arksey and O’Malley’s [27] scoping review framework was to collate and summarize the results for presentation and discussion. Each selected article was summarized in a customized data characterization utility form to guide data extraction. The goal of this step was to determine and chart factors to be extracted from each article to help answer the research question [26, 27, 30]. The charting of data was an iterative and exploratory process in which the data charts were continuously updated to ensure completeness and accuracy [26, 30]. Data extracted from the charts included year of publication, country of study, implications for policy and practice, types of ICT interventions, demographics, empowerment (definition, as a design consideration, and measures), and social determinants of health (presence and description within in the study). All data were then analyzed using thematic analysis and the main ideas refined over several iterations. The data were then mapped using tabular and visual presentations of the main conceptual categories followed by a narrative summary describing how the results related to the research question and objectives.

## Results

### Demographics and study characteristics

The geographic range of the included evidence was global; however, 41.1% (21/51) described research conducted in the USA. Seven studies were conducted in India, three in Australia, three in Sweden, and two in Canada. One study was conducted in each of the following countries: Finland, Ghana, Italy, Japan, Nepal, Netherlands, Nigeria, Singapore, South Korea, Sri Lanka, Tanzania, Thailand, Uganda, and United Kingdom.

Collation and comparison of demographics was difficult due to a lack of consistency in reporting. However, all articles described the demographics of women who were the primary focus of the study. Seventy eight percent of the articles (40/51) reported on some or all of the descriptive study characteristics. The age of participants was frequently reported although there were inconsistent age groupings across the studies. Some articles only reported the mean age of participants, while others provided only an age range. The lowest mean age reported was 24 years while the highest was 59.6 years; the categories ranged from less than 16 years to 64 years and older. It is difficult to compare these age ranges as the categories varied in the included articles, and it was unclear as to whether age was simply a descriptive statistic designed to describe the sample or whether it was reflected upon consistently in terms of the overall implications to the study.

Missing from the studies were the perspectives and participation of women who could not access, afford, and/or purchase an ICT device as well as effectively and fully utilize it to support their empowerment. Exclusion criteria used in the individual 51 studies illustrate that women not already owning a mobile device, computer, or tablet were eliminated from participating in the research.

### Empowerment definition

In the included studies, the concept of empowerment was used incongruously with terms like self-concept, self-esteem, and self-worth, sometimes by the same author in the same study, which further limited our ability to achieve a uniform definition for the purposes of this research. Less than one quarter (12/51) of the studies used the term “empower(ment)” in their definition of the concept of interest. These studies defined empowerment as a process but with different foci: as individuals having choice or control over their decisions [31–38], as being multi-dimensional and influencing a variety of areas [34, 37, 39, 40] or with a focus on building individuals’ capacities, including internal and external resources [39–42].

The remaining studies described empowerment in a more indirect way, never including the term “empower” or “disempower.” Instead, the term empowerment was described in synonymous terms, for instance, half (28/51) described the concept of empowerment as the process of enabling a sense of self-efficacy or self-worth in the ability to overcome barriers to resources, as well as the barriers to decision-making control [43–70]. One fifth (11/51) described empowerment as the process enabling a sense of self-efficacy or self-worth in the ability to overcome barriers to control over resources [71–81].

### Measures of empowerment

All studies considered the concept of empowerment in their design; 80% (41/51) of the articles considered empowerment as a primary outcome of the study. No measures of empowerment were specifically cited in any of the articles, beyond the measures of the behaviour being studied. Several studies included various measures of self-efficacy (i.e., childbirth [74], physical activity [56, 67, 71, 74, 80, 81], intimate partner violence [72], caregiving [75], barrier [55, 56], health [78], and chronic disease management [77]). The authors of the articles did not compare the different types of self-efficacy scales for validity of empowerment. The diversity of the scales illustrates a focus on improving efficacy of individual behaviors rather than the holistic empowerment of women.

### ICT interventions to support women’s capacity and tools

The articles described a range of supportive ICT interventions, though with inconsistent and overlapping classification. The specific types of interventions covered in the 51 articles included web-based devices (17), the internet (19), particular websites (3), blogs (1), text messaging (4), telemedicine (1), video (1), apps (5), social media (2), computers (6), email (1) and Fitbit™ (1). Our categorization of ICTs focused on how the specific interventions were utilized in the day to day lives of women and were obtained from a thematic analysis of the types of ICTs used by women in the studies. The themes included (1) Outreach; (2) Education; (3) Lifestyle (4) Health Challenges; (5) Prevention; and (6) Perception of Barriers.

### Outreach

Ten of the 51 articles reviewed described supportive ICT interventions as a means of outreach or connecting with clients in the community. Common themes in this section included supporting women where they are at in the community, in terms of their social position, to enhance positive health behaviours with technological assistance, as well as overall enhanced accessibility to ICTs. This was accomplished through Cognitive Behavioural Therapy using computers [46], and web-based decision aid for understanding fetal anomalies [47]. Educational text messages were sent to encourage breastfeeding [79], and general health promotion interventions were delivered as well [43, 44, 56, 65, 73, 75, 80].

### Education

Six articles described supportive ICT interventions that delivered various health information, through smartphones or other web-based devices. These included Facebook™ virtual learning systems [34], psychoeducation for breast cancer patients [35], as well as interactive voice response as a tool for improving access to healthcare in remote areas [59]. Other interventions included English language programs [70], antenatal perineal massage support groups [76], as well as support for enhancing doctor-patient relationships. [64].

### Lifestyle

Twelve articles described supportive ICT interventions that focused on behavioural outcomes related to general lifestyle areas, using web-based devices. Commonly, the interventions provided some form of external support for women to improve their overall way of being healthy. These included improving nutrition knowledge and behaviours [67, 69], promoting healthy food planning, shopping, and eating behaviours [54], interventions for weight loss behaviours, [45] and engagement with physical activity coaching [55, 71, 74]. Many of the interventions focused on social networks [9, 48], for example, peer support for building social capital [52], and promoting social behaviours through an iPad book club [81].

### Health challenges

Eleven articles described ICT interventions that focused on using web-based devices to address specific health challenges. The health challenges largely focusing on ways to enhance maintenance of women’s health, for example, self-paced education programs for those who experience intimate partner, as well as dating violence [33, 41, 72], and educational training to enhance understanding and management of chronic illness [77]. The interventions addressing health challenges were concentrated on those that affect women only, for example educational training for patients with breast cancer [38, 61], health modules for those with breast cancer [78] and stress incontinence [63] and advanced care planning for women with ovarian cancer [49].

### Prevention

A few articles (3/51) described ICT interventions that focused on preventing specific health challenges using web-based devices. One intervention focused on the prevention of sexual and reproductive illness using education information [57]. Another encouraged vaccination behaviors and immunization with educational information [58] as well as the prevention of pre-eclampsia in rural developing countries using diagnostic tools [51]. One study focused on utilizing mobile phones to manage money transfers to support transport of women with fistula to urban hospitals [60] and another examined electronic health records to improve breast cancer screening [53].

### Perceptions of barriers

Nine articles described ICT interventions that focused on the perception of barriers to ICTs that assist women in advancing their understanding and use of ICTs. These studies focused on the perceived barriers and understanding of the role of mobile phones, [42, 66] the awareness of gender-based barriers in telemedicine [68], the development of women through mobile phones [32, 40], as well as the connection with women in the community apps [50].

## Discussion

### Concept of empowerment

Empowerment is a multi-dimensional and contextual concept that is internal by nature, varies in meaning, and reflects how women self-ascribe it to themselves. From the outset of the review, search terms had to include words beyond simply “empower[ment]” as much of the initial searching revealed synonyms including self-efficacy, self-worth, self-concept, and/or capacity. This inconsistency in the use of the term empowerment yields a lack of consensus on how empowerment is understood which impacts how research studies and interventions are structured and delivered to ensure maximum effectiveness and generalizability. While none of the studies included in the review indicated the broader negative outcomes related to the use of ICT, the literature supports a flip side to using technology to empower women. For example, technological advances are disproportionately accompanied by female-directed cyber abuse [82, 83].

Evidence that women of poor socio-economic status are being left out of research studies and programs that aim to support women’s empowerment, highlights that targeted access and funding for at risk populations (such as sub-populations of women) are essential considerations in policy and program development across individual, community, and global contexts. This also reflects biases in terms of the population sub-groups in research studies that aim to advance empowerment. Opportunities exist for further evaluation of how empowerment is being measured and used in conjunction with ICTs, as well as which frameworks are being used to guide research in this area. The lack of specific measures of empowerment reflects a barrier, not only regarding how strategies for empowerment are understood and implemented, but how researchers know whether empowerment has been achieved. The finding underscores a need for a standardized tool for measuring the level of women’s empowerment.

### ICTs to improve empowerment

Empowerment through ICTs has the potential to cross multiple sectors, both private and public. The complexity of empowerment and ICTs, as they relate to the root issues of inequities, suggests the need for collaborative, multi-sectoral involvement. These partnerships consider the contextual factors that act as facilitators and barriers for women in all types of communities. Interagency partnerships are uniquely suited to develop interventions aimed at enabling women to make better use of ICTs. These interventions should include information on access to education, facilities for education regarding entrepreneurship, employment opportunities, and health and other government health resources. Governments partnering with private telecommunication agencies through subsidization could provide discounted or refurbished devices for women who are deemed disadvantaged. Funding may also benefit those who experience difficulty in obtaining mobile devices as well as in accessing interventions aimed at enhancing the use of ICT. For example, funding is needed to support the cost of accessing services, low-cost devices, or the provision of Subscriber Identity Module (SIM) cards. Alternatively, governments should support and encourage private mobile operators through tax exemptions and other benefits to facilitate better mobile services and infrastructure in rural, remote, and urban areas. Providing accessible computer sites within communities or in schools is another way to bridge the gap in access to and use of ICT. These strategies not only help in improving the overall status of girls and women but also influence overall empowerment and development of the community.

Though ICT is not the only factor that can support women’s empowerment through capacity building, women who do not have access to or who cannot afford ICTs, are potentially disempowered due to a lack of voice and participation within the information sphere. Exclusion of such women from research limits the measurement of the true impact of ICTs on empowerment and generalizability of findings. Continued research regarding empowerment involving more advantaged sub-groups of women does not address the inherent issues of oppression of women within society and further disempowers those under-represented groups. Local policies (such as affordable internet as a basic need; basic digital literacy education embedded in local curricula) have the greatest potential of improving the uptake of ICTs, as this process occurs initially at the individual level.

### Individual, community, and global knowledge

Local and national governments need to invest in information gathering tools that inquire how and why women are using technology to support their lives and families. Equally important is the inquiry of women’s perceptions regarding how they prefer to use ICTs to improve their lives or the barriers they experience in the process. A global survey undertaken by the UN Statistics Division in 2011 indicated that only 30 percent of countries regularly produce sex-disaggregated statistics (such as male:female access to ICT; digital literacy by gender) and existing data collection approaches do not incorporate qualitative components that highlight the voices of women [84].

Future data should be translated into gender sensitive policies that support equal access and use of ICTs. The development and implementation of such policies should involve representation of women from all socio-economic backgrounds and ages to ensure maximum impact. Examples include policies that allow women to effectively access and participate in ICTs within society, the delivery of ICTs at a reasonable cost for all, as well as policies that regulate the cost and provision of services linked to ICTs such as availability of cell phone, easily accessible WiFi sites, and cost-effective internet plans.

## Limitations

While scoping reviews examine the breadth of evidence available on a topic, they do not factor in the depth or quality of that evidence [25–27, 30]. Some authors have argued that scoping reviews should include an assessment of quality; however, Armstrong et al. [25] contend that this decision should depend on the resources available for the review as well as the purpose of the scoping review itself. The quantity of data that is generated in a scoping review can be significant and so it is important to find a balance between providing an overview of all types of evidence found and providing detailed data and assessment of a smaller number of studies [25]. Scoping studies also lack a thorough evaluation of the quality of results, instead producing a narrative account of all available evidence [26, 27]. This approach serves to ensure that all resulting evidence is included in the review and does not limit the end number of articles, as in a systematic review.

## Conclusions

The diversity of technological interventions utilized to support empowerment is infinite and there is no limit to how ICTs can be implemented in daily lives. This study is novel and essential as it comprehensively describes efforts to use ICTs to empower women, and the imperative for collaborations between researchers, program implementers and policy makers to address the persistent gender disparities in the access to and use of technologies. This research provides a foundation for future research on the concept of empowerment with ICTs in critical areas of outreach, education, lifestyle, health challenge, prevention, and perception of barriers. Outreach was linked to positive health behaviours such as health promotion and decision-making applications. Education interventions varied from learning systems to health relationships for knowledge sharing. Lifestyle ICT interventions were related to external supports, often peer based, for improving healthful choices such as coaching and planning tools. Health challenges and prevention were relevant to specific challenges (e.g., intimate partner violence; chronic diseases) and health literacy issues (e.g., vaccine awareness; screening programs), respectively. The final theme of perceptions of barriers reflected experiences by participants respecting uptake, utilization, and ubiquity of ICTs. Each of these areas is well situated for future intervention research and each area brings focal points and imperatives to this emerging research agenda.

## Data Availability

The databases used in the study were all open access and included Scopus, Embase, ABI Inform, Soc Index, Sociological Abstracts, Gender Studies, Springer Link, PsychInfo, Science Direct, and Academic Search Complete. The datasets used and/or analysed during the current study are available from the corresponding author on reasonable request.

## Abbreviations

SDG: Sustainable Development Goals
ICT: Information and communication technology
ICTs: Information and communications technologies
WHO: World Health Organization
UN: United Nations
ITU: International Telecommunication Union
GPS: Global Positioning System
SIM: Subscriber Identity Module
